# Elevated Circulating Adipocyte‐Fatty Acid Binding Protein Levels Predict Incident Cardiovascular Events in a Community‐Based Cohort: A 12‐Year Prospective Study

**DOI:** 10.1161/JAHA.112.004176

**Published:** 2013-02-22

**Authors:** Wing Sun Chow, Annette Wai Kwan Tso, Aimin Xu, Michele Mae Ann Yuen, Carol Ho Yi Fong, Tai Hing Lam, Su Vui Lo, Hung Fat Tse, Yu Cho Woo, Chun Yip Yeung, Bernard Man Yung Cheung, Karen Siu Ling Lam

**Affiliations:** 1 Department of Medicine, The University of Hong Kong, Hong Kong (W.S.C., A.W.K.T., A.X., M.M.A.Y., C.H.Y.F., H.F.T., Y.C.W., C.Y.Y., B.M.Y.C., K.S.L.L.); 2 Research Centre of Heart, Brain, Hormone and Healthy Aging, The University of Hong Kong, Hong Kong (A.W.K.T., A.X., H.F.T., B.M.Y.C., K.S.L.L.); 3 School of Public Health, The University of Hong Kong, Hong Kong (T.H.L.); 4 Hospital Authority, Hong Kong (S.V.L.)

**Keywords:** adipocyte‐fatty acid binding protein, adipokines, adiponectin, cardiovascular diseases, inflammation

## Abstract

**Background:**

Obesity is closely associated with various cardiovascular diseases (CVDs). Adipose tissue inflammation and perturbation of adipokine secretion may contribute to the pathogenesis of CVD. This study aimed to evaluate whether the 2 most abundant adipokines, adipocyte‐fatty acid binding protein (A‐FABP) and adiponectin, are independent risk factors predisposing to CVD.

**Method and Results:**

We investigated prospectively the 12‐year development of CVD in relation to the baseline levels of A‐FABP and adiponectin in a population‐based community cohort comprising 1847 Chinese subjects recruited from the Hong Kong Cardiovascular Risk Factors Prevalence Study 2 (CRISPS 2) cohort without previous CVD. Baseline serum levels of A‐FABP, adiponectin, and C‐reactive protein (CRP), an established biomarker predictive of CVD, were measured. In all, 182 (9.9%) of the 1847 Chinese subjects developed CVD during a median follow‐up of 9.4 years. The CVD group had more traditional risk factors, higher baseline levels of A‐FABP and CRP (both *P*<0.001), but similar adiponectin levels (*P*=0.881) compared with the non‐CVD group. In Cox regression analysis including both biomarkers, the adjusted HR for A‐FABP and CRP for subjects above the optimal cutoff values were 1.57 (95% CI, 1.14 to 2.16; *P*=0.006) and 1.60 (95% CI, 1.12 to 2.27; *P*=0.01), respectively, after adjustment for traditional risk factors. The category‐free net reclassification index, but not the c‐statistic, showed improvement in predictive performance by the addition of A‐FABP to the traditional risk factor model (*P*=0.017).

**Conclusions:**

Circulating A‐FABP level predicts the development of CVD after adjustment for traditional risk factors in a community‐based cohort. Its clinical use for CVD prediction warrants further validation.

## Introduction

Obesity is closely associated with various metabolic and cardiovascular diseases (CVDs), including insulin resistance, type 2 diabetes, hypertension, coronary heart disease, and stroke. Adipose tissue, apart from its traditional role as energy storage, acts as an endocrine organ through the production of adipokines, and thereby participates in the regulation of insulin sensitivity, lipid metabolism, and cardiovascular homeostasis.^1^ The majority of adipokines, including adipocyte‐fatty acid binding protein (A‐FABP), tumor necrosis factor alpha (TNF‐α), interleukin‐1β (IL‐1β), plasminogen activator inhibitor‐1, and resistin, possess proinflammatory properties, whereas others, such as adiponectin and adrenomedulin, have an anti‐inflammatory effect. A‐FABP and adiponectin are the 2 most abundant adipokines produced by adipocytes. Recent studies suggest that adipose tissue inflammation, characterized by the infiltration of inflammatory cells and perturbation of adipokine secretion, may play a central role in the pathogenesis of obesity‐related pathologies.^2^ A‐FABP is a cytoplasmic lipid chaperon and plays an important role in mediating intracellular fatty acid trafficking. In addition, A‐FABP may potentiate vascular inflammation and participate in the pathogenesis of CVD.^3–4^ In animal studies, A‐FABP deficiency resulted in a significant reduction of aortic atherosclerotic lesions in apolipoprotein‐E (apoE)–deficient mice, a mouse model with accelerated atherosclerosis, and a higher survival rate when they were fed on a high‐fat atherogenic diet.^5–6^ In contrast, the administration of recombinant adiponectin has been shown to protect against obesity‐related disorders, such as insulin resistance in obese diabetic (ob/ob) mice, hypertension in severely obese mice, dyslipidemia in mice with protease inhibitor–induced metabolic disorder, and atherosclerosis in apoE‐deficient mice.^7–10^ Furthermore, adiponectin‐knockout mice are more susceptible to ischemia‐induced myocardial infarction when compared with their wild‐type littermates.^11^ In line with these animal studies, hypoadiponectinemia has been demonstrated to be an independent risk factor for insulin resistance, hypertension, and myocardial infarction in humans.^12–14^ On the other hand, elevated circulating A‐FABP levels are closely associated with obesity, the number of components of metabolic syndrome, increased carotid intima‐media thickness, and ischemic stroke.^15–17^ In addition, we have shown in prospective studies that A‐FABP can predict the development of the metabolic syndrome and type 2 diabetes.^18–19^ A recent study also revealed that A‐FABP levels in atherosclerotic plaques were associated with an unstable plaque phenotype and were predictive of the occurrence of adverse CVD among subjects who underwent carotid endarterectomy.^20^ However, prospective studies linking circulating A‐FABP levels with cardiovascular outcomes in the general population are still lacking, and controversial results have been obtained from prospective studies addressing the relationship between serum adiponectin levels and incident CVD.^21–22^ To evaluate whether the circulating levels of these 2 most abundant adipokines are independent risk factors that predict CVD in humans, we investigated prospectively the 12‐year development of incident CVD in relation to the baseline levels in a population‐based community cohort comprising 1847 Chinese subjects recruited from the Hong Kong Cardiovascular Risk Factor Prevalence Study (CRISPS).^23^

## Methods

The Hong Kong CRISPS cohort is a population‐based prospective study with comprehensive cardiovascular risk assessment in Hong Kong Chinese. The study first commenced as a cross‐sectional survey in 1995–1996, conducted to assess the prevalence of cardiovascular risk factors in the Hong Kong population. In all, 2895 subjects (1412 men and 1483 women), aged 25 to 74, were recruited from the general population through random selections of telephone numbers. After baseline assessment at the Queen Mary Hospital, subjects were invited for prospective follow‐up to assess their development of major cardiovascular risk factors, including type 2 diabetes, hypertension, and dyslipidemia, with subsequent assessments carried out in 2000–2004 (median follow‐up of 6.4 years, CRISPS‐2).^24^ Subjects attended all assessments after an overnight fast. At each assessment, demographic data, including age, sex, occupation, smoking, alcohol consumption, and physical activity, were ascertained. Detailed medical, drug, and family histories, including history of CVD, were obtained using a detailed questionnaire. Anthropometric parameters, including body weight, height, body mass index (BMI), waist and hip circumferences, and blood pressure were measured as previously reported.^13,25^ All subjects except those on antidiabetic drugs underwent a 75‐g oral glucose tolerance test. Blood was also drawn for fasting lipid and insulin measurements. At CRISPS‐2, plasma and serum samples were stored in aliquots at −70°C for the measurement of A‐FABP, adiponectin, and C‐reactive protein (CRP). As only 644 of these stored samples were available from CRISPS‐1, assessments of the relationship between these biochemical parameters and incident CVD were done using CRISPS‐2 as baseline, and after excluding those subjects with known CVD. Incident CVD events since CRISPS‐2 were evaluated during 2000–2011 (median follow‐up of 9.4 years). Measurements of the biochemical parameters including glucose, insulin, and lipid levels were described previously.^18,26^ A‐FABP was measured using an ELISA (BioVendor Laboratory Medicine, Modrice, Czech Republic).^18^ Adiponectin was measured with an in‐house sandwich ELISA established in our laboratory.^13,27^ CRP was measured with a high‐sensitivity, particle‐enhanced immunoturbidimetric assay (Roche Diagnostics, GmbH, Mannheim, Germany).

Diabetes was defined as being on antidiabetic drugs or fulfilling the diagnostic criteria for diabetes according to the World Health Organization 1998 diagnostic classification.^28^ Hypertension was defined as having a sitting blood pressure ≥140/90 mm Hg or on regular antihypertensive drugs, and dyslipidemia was defined as having ≥1 of the following criteria: (1) triglycerides (TG) ≥1.7 mmol/L; (2) HDL‐cholesterol <1.04 mmol/L in men and <1.29 mmol/L in women; (3) LDL‐cholesterol ≥3.4 mmol/L; and (4) already on lipid‐lowering drugs. Diagnoses of CVD events, based on the ICD‐9 (402, 404, 410‐414, 425‐447, 518.4), which included, among others, acute myocardial infarction, angina pectoris, stroke, and heart failure, were verified from the Hospital Authority database or its private practitioners in 2011, including the dates of the events and the discharge diagnosis. For subjects who had died, causes and dates of death were ascertained from the Hong Kong Death Registry. The medical diagnoses were reviewed by 2 physicians independently; disagreements between them were resolved by a third. The concordance between the 2 physicians was 0.98. This study was approved by the Institutional Review Board of the University of Hong Kong/Hospital Authority Hong Kong West Cluster, and all participants gave written informed consent.

### Statistical Analysis

All analyses were performed with SPSS Statistics 19 (SPSS, Chicago, IL) and R‐programming language, version 2.14.2. Results were presented as mean±SD or median with interquartile range (IQR) as appropriate. For data that were not normally distributed, natural logarithmic transformation was applied before analyses. Variables were compared between groups by 1‐way ANOVA for continuous data and chi‐square or Fisher's exact test for categorical data as appropriate. Partial correlation was used to examine the associations between biochemical parameters and various anthropometric measurements. The optimal cutoff values for A‐FABP and CRP were derived using Youden's j criterion.^29^ Optimal cutoff points were derived from the Youden index criterion (maximum of [sensitivity+specificity−1]). Cox proportional hazards regression (Cox regression) was used to estimate the hazard ratios (HRs) and 95% CIs for incident CVD after adjustment of the traditional risk factors including age, sex, BMI, smoking status, diabetes, hypertension, and dyslipidemia. Assumption of proportional hazard was validated for all covariates (global test) and, variable by variable, using scaled Schoenfeld residuals. According to these tests, all Cox regression models did not violate the assumption (*P*>0.05). In addition, a propensity score–based approach was undertaken to control for baseline traditional risk factors in the logistic regression model. Subjects were then stratified into quintiles according to their predicted propensity score. The Mantel–Haenszel odds ratio for biomarkers was estimated. The predictive performances of various models were assessed by receiver operating characteristic (ROC), category‐free net reclassification improvement (NRI), and integrated discrimination improvement (IDI). Comparison between 2 areas under the ROC curves (AUCs) was examined using the DeLong method.^30^ Interrater reliability was determined by Cohen's kappa. Two‐sided *P* values <0.05 were considered statistically significant.

## Results

Of 2895 subjects who participated in CRISPS‐1, 1944 returned for assessment at CRISPS‐2. After excluding 69 subjects with known CVD and 28 subjects with missing data, there were 1847 subjects followed for a median duration of 9.4 years, 182 of whom developed CVD (9.9%; incidence rate, 10.89 per 1000 person‐years). Subjects with incident CVD (CVD group) were older, more likely to be male, and more likely to be hypertensive (57.7% versus 22.5%), diabetic (28.6% versus 13.5%), dyslipidemic (77.5% versus 62.0%), and a current/former smoker (42.3% versus 24.2%) at baseline (all *P*<0.001). They had higher BMI, waist circumference (WC), systolic and diastolic blood pressure (SBP and DBP, respectively), fasting glucose (FG) levels, and greater insulin resistance as measured by homeostasis model assessment–insulin resistance (HOMA‐IR; all *P*<0.001 except for DBP, *P*=0.003), compared with those who did not develop CVD events (non‐CVD group) (Table 1). They also had higher TG and lower HDL‐cholesterol levels (both *P*<0.001), but similar LDL‐cholesterol levels (*P*=0.081). Of the biomarkers, the CVD group had higher baseline CRP (*P*<0.001) and A‐FABP (sex‐adjusted *P*<0.001), but similar adiponectin levels (sex‐adjusted *P*=0.881) compared with the non‐CVD group (Table 1).

**Table 1.** Baseline Characteristics of Subjects With and Without Incident CVD After a Median Follow‐Up of 9.4 Years

	CVD	Non‐CVD	*P* Value
n	182	1665	—
Sex, % men	64.8	43.9	<0.001
Age, y	62.9±10.9	50.9±11.4	<0.001
Smoking status, %	42.3	24.2	<0.001
Body mass index, kg/m^2^	25.2±3.54	24.0±3.52	<0.001
Waist circumference, cm			<0.001*
Men	87.4±10.2	83.8±8.81	
Women	82.8±8.40	75.8±9.02	
Fasting glucose, mmol/L*	5.56±1.02	5.20±0.95	<0.001
HOMA‐IR**	2.12 (1.36 to 3.23)	1.62 (1.14 to 2.41)	<0.001
Diabetes, %	28.6	13.5	<0.001
Systolic blood pressure, mm Hg*	134.2±18.9	119.2±17.0	<0.001
Diastolic blood pressure, mm Hg*	77.3±11.4	74.5±10.3	0.003
Hypertension, %	57.7	22.5	<0.001
HDL‐cholesterol, mmol/L*	1.26±0.35	1.41±0.37	<0.001
LDL‐cholesterol, mmol/L*	3.40±0.87	3.29±0.81	0.081
Triglycerides, mmol/L**	1.30 (0.90 to 1.80)	1.10 (0.80 to 1.60)	<0.001
Dyslipidemia, %	77.5	62.0	<0.001
C‐reactive protein, mg/L*	1.28 (0.63 to 2.42)	0.68 (0.33 to 1.47)	<0.001
Adiponectin, μg/mL*			0.881*
Men	5.72 (3.84 to 8.54)	5.51 (3.59 to 8.61)	
Women	6.97 (5.18 to 10.6)	7.82 (5.36 to 11.5)	
A‐FABP, μg/L*			<0.001*
Men	22.3 (16.9 to 33.2)	18.6 (13.7 to 24.7)	
Women	33.9 (25.3 to 44.0)	23.4 (17.0 to 32.2)	

Data are expressed as mean±SD or median (interquartile range). CVD indicates cardiovascular disease; HOMA‐IR, homeostasis model assessment–insulin resistance; HDL, high‐density lipoprotein; LDL, low‐density lipoprotein; A‐FABP, adipocyte‐fatty acid binding protein.

* Sex‐adjusted *P* value.

* Excluded n=89 subjects on antidiabetic drugs.

* Excluded n=186 subjects on antihypertensive drugs.

* Excluded n=44 subjects on lipid‐lowering drugs.

* Log‐transformed before analysis.

The partial correlations among various biomarkers and baseline characteristics are shown in Table 2. A‐FABP and CRP showed direct correlations with traditional CVD risk factors, including BMI, WC, FG, HOMA‐IR, SBP, DBP, LDL‐cholesterol, and TG (A‐FABP: all *P*<0.001 except for FG, *P*=0.002; CRP: all *P*<0.001 except for LDL‐cholesterol, *P*=0.001) and inverse correlations with HDL‐cholesterol (both *P*<0.001). In contrast, adiponectin was negatively correlated with these risk factors (all *P*<0.001 except for LDL‐cholesterol, *P*=0.015), and positively correlated with HDL‐cholesterol (*P*<0.001). Among the biomarkers, A‐FABP showed an inverse correlation with adiponectin (*r*=−0.15, *P*<0.001), but a direct correlation with CRP (*r*=0.23, *P*<0.001), whereas adiponectin was negatively correlated with CRP (*r*=−0.21, *P*<0.001), with all *P* values being age‐ and sex‐adjusted (Table 2).

**Table 2.** Partial Correlations Among Various Biomarkers and Baseline Characteristics

Baseline Parameters	Age‐ and Sex‐Adjusted *r*,* P* Value		
	A‐FABP*	Adiponectin*	C‐Reactive Protein*
Body mass index	0.44, <0.001	−0.33, <0.001	0.38, <0.001
Waist circumference	0.42, <0.001	−0.33, <0.001	0.35, <0.001
Fasting glucose*	0.07, 0.002	−0.20, <0.001	0.10, <0.001
HOMA‐IR**	0.30, <0.001	−0.37, <0.001	0.27, <0.001
Systolic blood pressure*	0.15, <0.001	−0.12, <0.001	0.18, <0.001
Diastolic blood pressure*	0.20, <0.001	−0.13, <0.001	0.18, <0.001
HDL‐cholesterol*	−0.22, <0.001	0.40, <0.001	−0.27, <0.001
LDL‐cholesterol*	0.15, <0.001	−0.06, 0.015	0.08, 0.001
Triglycerides**	0.32, <0.001	−0.34, <0.001	0.23, <0.001
Adiponectin*	−0.15, <0.001	—	—
C‐reactive protein*	0.23, <0.001	−0.21, <0.001	—

A‐FABP indicates adipocyte‐fatty acid binding protein; HOMA‐IR, homeostasis model assessment–insulin resistance; HDL, high‐density lipoprotein; LDL, low‐density lipoprotein.

* Log‐transformed before analysis.

* Excluded n=89 subjects on antidiabetic drugs.

* Excluded n=186 subjects on antihypertensive drugs.

* Excluded n=44 subjects on lipid‐lowering drugs.

Using Cox regression analysis, the HR for a 1‐unit increase in log A‐FABP and CRP in predicting 9.4‐year incident CVD was 1.50 (95% CI, 1.04 to 2.15; *P*=0.029) and 1.30 (95% CI, 1.12 to 1.52; *P*=0.001), respectively, after adjustment for traditional risk factors (Table 3). Analysis using continuous lipid variables revealed similar findings (data not shown). When expressed as continuous variables, A‐FABP was not predictive of CVD in the presence of CRP (*P*=0.064, Table 3, model 3). Of the traditional risk factors, age, sex (male), and hypertension were significant independent risk factors in all 3 models (all *P*≤0.001), whereas smoking (current/former smoker) was significant only in model 2 (*P*<0.05). There was no sex interaction between A‐FABP and incident CVD (*P*=0.157). In addition, both NRI (18.6% [3.3% to 33.9%]; *P*=0.017) and IDI (0.25% [0.17% to 0.46%]; *P*=0.016) were statistically significant, demonstrating improvement in the predictive performance by the addition of A‐FABP to the traditional risk factor model, although a similar finding could not be shown using the minimal differences of area under the curve (*P*=0.837) (Table 4).

**Table 3.** Multivariable Cox Proportional Hazards Regression Showing Significant Predictors of Incident CVD After Adjustment for Traditional Risk Factors

Variables	Model 1		Model 2		Model 3	
	HR (95% CI)	*P* Value	HR (95% CI)	*P* Value	HR (95% CI)	*P* Value
A‐FABP*	—	—	**1.50 (1.04 to 2.15)**	**0.029**	1.42 (0.98 to 2.04)	0.064
C‐reactive protein*	**1.30 (1.12 to 1.52)**	**0.001**	—	—	**1.29 (1.10 to 1.50)**	**0.001**

Models were adjusted for the traditional risk factors including age, sex, BMI, smoking status, diabetes, hypertension, and dyslipidemia. CVD indicates cardiovascular disease; HR, hazard ratio; CI, confidence interval; A‐FABP, adipocyte‐fatty acid binding protein. Bold used to highlight those p‐values<0.05.

* Log‐transformed before analysis.

**Table 4.** Discrimination and Reclassification of Incident CVD With Various Prediction Multivariable Cox Regression Models

Old Model	New Model	AUC (95% CI)	DeLong *P* Value	NRI (95%CI)	*P* Value	IDI (95% CI)	*P* Value
Traditional risk factors	—	0.819 (0.801 to 0.837)	—	**—**	**—**	**—**	**—**
Traditional risk factors	+A‐FABP	0.820 (0.802 to 0.837)	0.837	**18.6% (3.3 to 33.9)**	**0.017**	**0.25% (0.17 to 0.46)**	**0.016**
Traditional risk factors	+CRP	0.824 (0.806 to 0.837)	0.184	**22.1% (6.8 to 37.4)**	**0.005**	**0.42% (0.12 to 0.72)**	**0.006**
Traditional risk factors+CRP	+A‐FABP	0.825 (0.806 to 0.842)	0.838	14.1% (−1.3 to 29.4)	0.072	**0.20% (0.02 to 0.37)**	**0.029**

Traditional risk factors included age, sex, body mass index, smoking status, diabetes, hypertension, and dyslipidemia; biomarkers were log‐transformed before analysis. CVD indicates cardiovascular diseases AUC, area under the curve; CI, confidence interval; NRI, net reclassification improvement; IDI, integrated discrimination improvement; A‐FABP, adipocyte‐fatty acid binding protein; CRP, C‐reactive protein. Bold used to highlight those p‐values<0.05.

**Figure 1.**
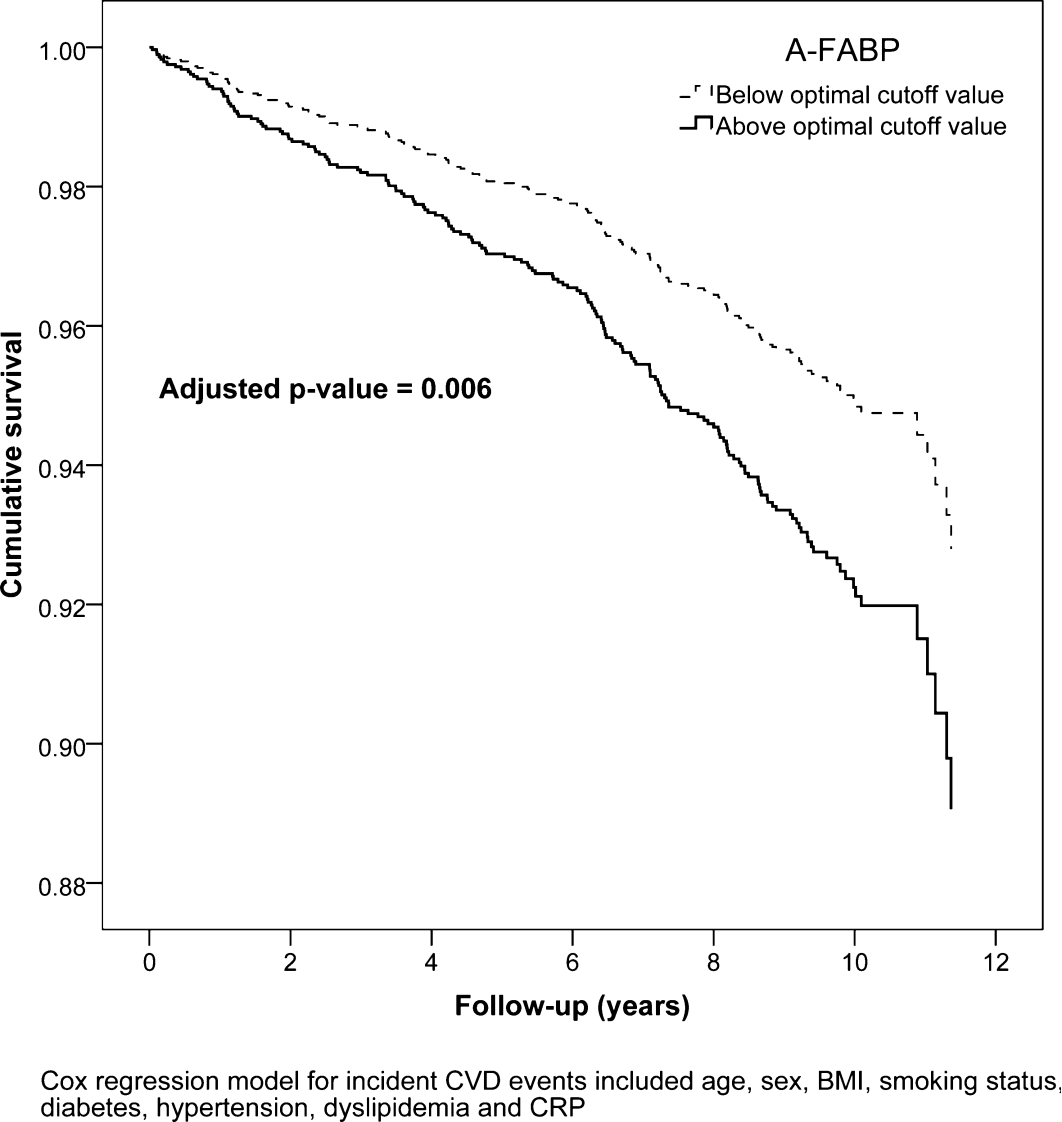
Cumulative survival curve of incident CVD over 12 years, based on the Cox proportional hazards model, in subjects above and below the optimal cutoff values of A‐FABP. CVD indicates cardiovascular disease; A‐FABP, adipocyte‐fatty acid binding protein; BMI, body mass index; CRP, C‐reactive protein.

Based on the highest Youden's j, the optimal cutoff value for A‐FABP was 26.2 μg/L for men and 30.2 μg/L for women and for CRP was 1.0 mg/L. Using the optimal cutoff values in the Cox regression analysis, A‐FABP, even after adjustment for CRP and traditional risk factors, predicted incident CVD (adjusted HR, 1.57 [1.14 to 2.16]; *P*=0.006; and 1.60 [1.12 to 2.27]; *P*=0.01 for A‐FABP and CRP, respectively). The cumulative survival curves for incident CVD, based on the Cox proportional hazards model and stratified by the optimal cutoff values of A‐FABP, are shown in Figure 1. Using propensity scores to control for traditional risk factors also revealed similar findings (data not shown).

## Discussion

In this prospective study, we found that an elevated circulating level of either A‐FABP or CRP could independently predict the development of CVD among men and women without previous CVD over and above the prediction based on traditional risk factors. Although the impact on the overall predictive performance by adding A‐FABP to a multivariable‐adjusted model was modest, our findings would support an association between A‐FABP and the development of obesity‐related CVD. To our knowledge, this is the first long‐term prospective study demonstrating that circulating A‐FABP level is linked to clinical cardiovascular outcomes in the general population. The attenuation of the predictive power of A‐FABP after adjustment for CRP when both were expressed as continuous variables may suggest that the pathogenic effect of A‐FABP is in part mediated through subclinical systemic chronic inflammation, of which CRP is the most established circulating biomarker. Nonetheless, the promising results of our subsequent analysis using optimal cutoff values by c‐statistics suggest that further large‐scale studies involving other long‐term follow‐up cohorts are warranted to establish A‐FABP as another biomarker for the clinical prediction of CVD outcome. For circulating adiponectin level, there was no statistically significant difference between CVD and non‐CVD groups, even by comparing the age‐adjusted level in the male subcohort, which had a higher event rate. The lack of a significant association between circulating adiponectin and incident CVD is in agreement with the finding of a meta‐analysis on the prospective relationship between adiponectin and CVD.^21^

CRP, produced mostly by hepatocytes in response to cytokines such as IL‐6 and TNF‐α, is the most extensively studied inflammatory marker in the literature. Koenig et al^31^ in the population‐based Monitoring of Trends and Determinants in Cardiovascular Disease (MONICA) Study, demonstrated that CRP enhanced global coronary risk, as assessed by the Framingham Risk Score. In their cohort, 191 (5.6%) of 3435 subjects developed events during an average follow‐up of 6.6 years. Subjects with CRP levels ≥3.0 mg/L had an adjusted hazard ratio of 2.21 (95% CI, 1.49 to 3.27), when compared with subjects with levels <1.0 mg/L. Our study revealed an adjusted hazard ratio of 1.60 (95% CI, 1.12 to 2.27) when comparing subjects with high or low CRP levels, using a cutoff value of 1.0 mg/L. It should be noted that our study population had a low cardiovascular risk, with the exclusion of known CVD subjects, and only 182 (9.9%) of 1847 subjects (incidence rate, 10.89 per 1000 person‐years) developed CVD over a median follow‐up of 9.4 years. Consistent with the low cardiovascular risk, our cohort had relatively low CRP levels (CVD/non‐CVD group: 1.28 mg/L [0.63 to 2.42 mg/L]/0.68 mg/L [0.33 to 1.47 mg/L]). Hence, our data suggest that CRP level is also useful in predicting CVD even among low‐risk subjects, when used in conjunction with traditional risk factors.

A‐FABP, also known as FABP4 and adipocyte fatty acid binding protein (aP2), is one of the most abundant proteins in mature adipocytes and is also expressed in macrophages.^32–33^ Although A‐FABP was originally identified as a cytoplasmic protein, we and others have shown that it is abundantly present in human serum and is increased with age and obesity.^15,34^ Apart from its correlations with various CVD risk factors, elevated circulating levels of A‐FABP have been associated with several markers of CVD, such as carotid intima‐media thickness, coronary atherosclerotic burden as measured by intravascular ultrasound, and the number of stenotic coronary arteries.^16,35–36^ In addition to its circulating levels, A‐FABP levels in carotid atherosclerotic plaques have been demonstrated to be associated with an unstable plaque phenotype.^20^ After a mean follow‐up of 2.4 years, subjects with elevated A‐FABP plaque levels showed a 2‐fold increased risk of reaching the primary outcome, defined as the composite of vascular death, vascular event, and surgical or percutaneous vascular intervention. We have previously demonstrated that high serum A‐FABP levels are independently associated with diabetic nephropathy and diabetic macrovascular diseases,^37^ and a recent study showed that serum A‐FABP predicts CV events in end‐stage renal disease.^38^ Furthermore, Tuncman et al^39^ demonstrated that the presence of a genetic variant at the A‐FABP promoter region that results in decreased A‐FABP expression was associated with reduced risk for CVD. Whereas the above studies are supportive of a role of A‐FABP in the pathogenesis of CVD in humans, this is the first outcome study demonstrating that elevated circulating A‐FABP levels can predict incident CVD on long‐term follow‐up of a community‐based cohort of relatively low CVD risk. Our results also show that the predictive power of A‐FABP can be attenuated after adjustment for CRP, which is consistent with the current knowledge of a close pathogenic link between A‐FABP and subclinical systemic chronic inflammation.^40^ In a murine macrophage cell line, expression of A‐FABP was increased on toll‐like receptor activation, whereas an orally active A‐FABP inhibitor significantly reduced the expression of several inflammatory cytokines, including TNF‐α and IL‐1β in macrophages, and decreased the atherosclerotic lesion area in mouse models.^33,41^ Nevertheless, our analysis could only demonstrate the close correlations among A‐FABP, CRP, and incident CVD but provide no evidence for any causal relationships.

Our study was limited by the low incident CVD event rate in our cohort, which precluded sex‐specific subgroup analyses of A‐FABP and adiponectin in predicting CVD. The test for sex interaction was also underpowered because of the low event rate among the female participants. We included only community‐based Chinese subjects without previous CVD, and therefore our findings could not be extrapolated to other ethnic groups or those subjects with higher CVD risk. Interestingly, during the revision of this article, we found that von Eynatten et al^42^ recently reported the association of an increase in serum A‐FABP level with fatal CVD outcomes over 10 years in subjects with known coronary heart disease. It is noteworthy that their cohort had a 2‐fold higher incidence rate than ours (24.00 versus 10.89 per 1000 person‐years). Taken together, these findings strongly support the role of A‐FABP in mediating CVD in humans, although our study design precluded the investigation of its direct causal relationship. To determine this causal relationship, long‐term interventional studies involving therapeutic agents that reduce A‐FABP expression or action such as A‐FABP inhibitors are warranted.^41^

In conclusion, we have demonstrated that an elevated circulating level of A‐FABP above the optimal cutoff value can potentially serve as a biomarker for the prediction of CVD, as an adjunct to traditional risk factors and established biomarkers such as CRP. These findings support the role of A‐FABP as a possible mediator of obesity‐related CVD, possibly by coupling lipid abnormality to subclinical inflammation.

## References

[1] Tilg H Moschen AR Adipocytokines: mediators linking adipose tissue, inflammation and immunity. Nat Rev Immunol. 2006; 6:772-783 16998510 10.1038/nri1937

[2] Li FY Cheng KK Lam KS Vanhoutte PM Xu A Cross‐talk between adipose tissue and vasculature: role of adiponectin. Acta Physiol (Oxf). 2011; 203:167-180 21062420 10.1111/j.1748-1716.2010.02216.x

[3] Hui X Li H Zhou Z Lam KS Xiao Y Wu D Ding K Wang Y Vanhoutte PM Xu A Adipocyte fatty acid‐binding protein modulates inflammatory responses in macrophages through a positive feedback loop involving c‐Jun NH2‐terminal kinases and activator protein‐1. J Biol Chem. 2010; 285:10273-10280 20145251 10.1074/jbc.M109.097907 PMC2856232

[4] Xu A Vanhoutte PM Adiponectin and adipocyte fatty acid binding protein in the pathogenesis of cardiovascular disease. Am J Physiol Heart Circ Physiol. 2012; 302:H1231-H1240 22210749 10.1152/ajpheart.00765.2011

[5] Makowski L Boord JB Maeda K Babaev VR Uysal KT Morgan MA Parker RA Suttles J Fazio S Hotamisligil GS Linton MF Lack of macrophage fatty‐acid‐binding protein aP2 protects mice deficient in apolipoprotein E against atherosclerosis. Nat Med. 2001; 7:699-705 11385507 10.1038/89076 PMC4027052

[6] Boord JB Maeda K Makowski L Babaev VR Fazio S Linton MF Hotamisligil GS Combined adipocyte‐macrophage fatty acid‐binding protein deficiency improves metabolism, atherosclerosis, and survival in apolipoprotein E‐deficient mice. Circulation. 2004; 110:1492-1498 15353487 10.1161/01.CIR.0000141735.13202.B6 PMC4027050

[7] Berg AH Combs TP Du X Brownlee M Scherer PE The adipocyte‐secreted protein Acrp30 enhances hepatic insulin action. Nat Med. 2001; 7:947-953 11479628 10.1038/90992

[8] Ohashi K Kihara S Ouchi N Kumada M Fujita K Hiuge A Hibuse T Ryo M Nishizawa H Maeda N Maeda K Shibata R Walsh K Funahashi T Shimomura I Adiponectin replenishment ameliorates obesity‐related hypertension. Hypertension. 2006; 47:1108-1116 16651465 10.1161/01.HYP.0000222368.43759.a1

[9] Xu A Yin S Wong L Chan KW Lam KS Adiponectin ameliorates dyslipidemia induced by the human immunodeficiency virus protease inhibitor ritonavir in mice. Endocrinology. 2004; 145:487-494 14592951 10.1210/en.2003-1140

[10] Okamoto Y Kihara S Ouchi N Nishida M Arita Y Kumada M Ohashi K Sakai N Shimomura I Kobayashi H Terasaka N Inaba T Funahashi T Matsuzawa Y Adiponectin reduces atherosclerosis in apolipoprotein E‐deficient mice. Circulation. 2002; 106:2767-2770 12451000 10.1161/01.cir.0000042707.50032.19

[11] Tao L Gao E Jiao X Yuan Y Li S Christopher TA Lopez BL Koch W Chan L Goldstein BJ Ma XL Adiponectin cardioprotection after myocardial ischemia/reperfusion involves the reduction of oxidative/nitrative stress. Circulation. 2007; 115:1408-1416 17339545 10.1161/CIRCULATIONAHA.106.666941

[12] Weyer C Funahashi T Tanaka S Hotta K Matsuzawa Y Pratley RE Tataranni PA Hypoadiponectinemia in obesity and type 2 diabetes: close association with insulin resistance and hyperinsulinemia. J Clin Endocrinol Metab. 2001; 86:1930-1935 11344187 10.1210/jcem.86.5.7463

[13] Chow WS Cheung BM Tso AW Xu A Wat NM Fong CH Ong LH Tam S Tan KC Janus ED Lam TH Lam KS Hypoadiponectinemia as a predictor for the development of hypertension: a 5‐year prospective study. Hypertension. 2007; 49:1455-1461 17452504 10.1161/HYPERTENSIONAHA.107.086835

[14] Kumada M Kihara S Sumitsuji S Kawamoto T Matsumoto S Ouchi N Arita Y Okamoto Y Shimomura I Hiraoka H Nakamura T Funahashi T Matsuzawa Y Association of hypoadiponectinemia with coronary artery disease in men. Arterioscler Thromb Vasc Biol. 2003; 23:85-89 12524229 10.1161/01.atv.0000048856.22331.50

[15] Xu A Wang Y Xu JY Stejskal D Tam S Zhang J Wat NM Wong WK Lam KS Adipocyte fatty acid‐binding protein is a plasma biomarker closely associated with obesity and metabolic syndrome. Clin Chem. 2006; 52:405-413 16423904 10.1373/clinchem.2005.062463

[16] Yeung DC Xu A Cheung CW Wat NM Yau MH Fong CH Chau MT Lam KS Serum adipocyte fatty acid‐binding protein levels were independently associated with carotid atherosclerosis. Arterioscler Thromb Vasc Biol. 2007; 27:1796-1802 17510463 10.1161/ATVBAHA.107.146274

[17] Tso AW Lam TK Xu A Yiu KH Tse HF Li LS Law LS Cheung BM Cheung RT Lam KS Serum adipocyte fatty acid‐binding protein associated with ischemic stroke and early death. Neurology. 2011; 76:1968-1975 21562251 10.1212/WNL.0b013e31821e54b3

[18] Xu A Tso AW Cheung BM Wang Y Wat NM Fong CH Yeung DC Janus ED Sham PC Lam KS Circulating adipocyte‐fatty acid binding protein levels predict the development of the metabolic syndrome: a 5‐year prospective study. Circulation. 2007; 115:1537-1543 17389279 10.1161/CIRCULATIONAHA.106.647503

[19] Tso AW Xu A Sham PC Wat NM Wang Y Fong CH Cheung BM Janus ED Lam KS Serum adipocyte fatty acid binding protein as a new biomarker predicting the development of type 2 diabetes: a 10‐year prospective study in a Chinese cohort. Diabetes Care. 2007; 30:2667-2672 17620449 10.2337/dc07-0413

[20] Peeters W de Kleijn DP Vink A van de Weg S Schoneveld AH Sze SK van der Spek PJ de Vries JP Moll FL Pasterkamp G Adipocyte fatty acid binding protein in atherosclerotic plaques is associated with local vulnerability and is predictive for the occurrence of adverse cardiovascular events. Eur Heart J. 2011; 32:1758-1768 21059735 10.1093/eurheartj/ehq387

[21] Sattar N Wannamethee G Sarwar N Tchernova J Cherry L Wallace AM Danesh J Whincup PH Adiponectin and coronary heart disease: a prospective study and meta‐analysis. Circulation. 2006; 114:623-629 16894037 10.1161/CIRCULATIONAHA.106.618918

[22] Wilson SR Sabatine MS Wiviott SD Ray KK De Lemos JA Zhou S Rifai N Cannon CP Morrow DA Assessment of adiponectin and the risk of recurrent cardiovascular events in patients presenting with an acute coronary syndrome: observations from the Pravastatin Or atorVastatin Evaluation and Infection Trial‐Thrombolysis in Myocardial Infarction 22 (PROVE IT‐TIMI 22). Am Heart J. 2011; 161:1147-1155 21641362 10.1016/j.ahj.2011.02.014

[23] Janus ED Epidemiology of cardiovascular risk factors in Hong Kong. Clin Exp Pharmacol Physiol. 1997; 24:987-988 9406673 10.1111/j.1440-1681.1997.tb02736.x

[24] Cheung BM Wat NM Man YB Tam S Thomas GN Leung GM Cheng CH Woo J Janus ED Lau CP Lam TH Lam KS Development of diabetes in Chinese with the metabolic syndrome: a 6‐year prospective study. Diabetes Care. 2007; 30:1430-1436 17337491 10.2337/dc06-1820

[25] Yeung DC Wang Y Xu A Cheung SC Wat NM Fong DY Fong CH Chau MT Sham PC Lam KS Epidermal fatty‐acid‐binding protein: a new circulating biomarker associated with cardio‐metabolic risk factors and carotid atherosclerosis. Eur Heart J. 2008; 29:2156-2163 18603624 10.1093/eurheartj/ehn295

[26] Wat NM Lam TH Janus ED Lam KS Central obesity predicts the worsening of glycemia in southern Chinese. Int J Obes Relat Metab Disord. 2001; 25:1789-1793 11781759 10.1038/sj.ijo.0801834

[27] Wang Y Lam KS Xu JY Lu G Xu LY Cooper GJ Xu A Adiponectin inhibits cell proliferation by interacting with several growth factors in an oligomerization‐dependent manner. J Biol Chem. 2005; 280:18341-18347 15734737 10.1074/jbc.M501149200

[28] Alberti KG Zimmet PZ Definition, diagnosis and classification of diabetes mellitus and its complications. Part 1: diagnosis and classification of diabetes mellitus provisional report of a WHO consultation. Diabet Med. 1998; 15:539-553 9686693 10.1002/(SICI)1096-9136(199807)15:7<539::AID-DIA668>3.0.CO;2-S

[29] Perkins NJ Schisterman EF The inconsistency of “optimal” cutpoints obtained using two criteria based on the receiver operating characteristic curve. Am J Epidemiol. 2006; 163:670-675 16410346 10.1093/aje/kwj063 PMC1444894

[30] DeLong ER DeLong DM Clarke‐Pearson DL Comparing the areas under two or more correlated receiver operating characteristic curves: a nonparametric approach. Biometrics. 1988; 44:837-845 3203132

[31] Koenig W Lowel H Baumert J Meisinger C C‐reactive protein modulates risk prediction based on the Framingham Score: implications for future risk assessment: results from a large cohort study in southern Germany. Circulation. 2004; 109:1349-1353 15023871 10.1161/01.CIR.0000120707.98922.E3

[32] Coe NR Bernlohr DA Physiological properties and functions of intracellular fatty acid‐binding proteins. Biochim Biophys Acta. 1998; 1391:287-306 9555061 10.1016/s0005-2760(97)00205-1

[33] Kazemi MR McDonald CM Shigenaga JK Grunfeld C Feingold KR Adipocyte fatty acid‐binding protein expression and lipid accumulation are increased during activation of murine macrophages by toll‐like receptor agonists. Arterioscler Thromb Vasc Biol. 2005; 25:1220-1224 15705927 10.1161/01.ATV.0000159163.52632.1b

[34] Stejskal D Karpisek M Adipocyte fatty acid binding protein in a Caucasian population: a new marker of metabolic syndrome? Eur J Clin Invest. 2006; 36:621-625 16919044 10.1111/j.1365-2362.2006.01696.x

[35] Miyoshi T Onoue G Hirohata A Hirohata S Usui S Hina K Kawamura H Doi M Kusano KF Kusachi S Ninomiya Y Serum adipocyte fatty acid‐binding protein is independently associated with coronary atherosclerotic burden measured by intravascular ultrasound. Atherosclerosis. 2010; 211:164-169 20193950 10.1016/j.atherosclerosis.2010.01.032

[36] Rhee EJ Lee WY Park CY Oh KW Kim BJ Sung KC Kim BS The association of serum adipocyte fatty acid‐binding protein with coronary artery disease in Korean adults. Eur J Endocrinol. 2009; 160:165-172 19001529 10.1530/EJE-08-0665

[37] Yeung DC Xu A Tso AW Chow WS Wat NM Fong CH Tam S Sham PC Lam KS Circulating levels of adipocyte and epidermal fatty acid‐binding proteins in relation to nephropathy staging and macrovascular complications in type 2 diabetic patients. Diabetes Care. 2009; 32:132-134 18931100 10.2337/dc08-1333 PMC2606847

[38] Furuhashi M Ishimura S Ota H Hayashi M Nishitani T Tanaka M Yoshida H Shimamoto K Hotamisligil GS Miura T Serum fatty acid‐binding protein 4 is a predictor of cardiovascular events in end‐stage renal disease. PLoS ONE. 2011; 6:e27356 22102888 10.1371/journal.pone.0027356 PMC3213139

[39] Tuncman G Erbay E Hom X De Vivo I Campos H Rimm EB Hotamisligil GS A genetic variant at the fatty acid‐binding protein aP2 locus reduces the risk for hypertriglyceridemia, type 2 diabetes, and cardiovascular disease. Proc Natl Acad Sci USA. 2006; 103:6970-6975 16641093 10.1073/pnas.0602178103 PMC1447594

[40] Makowski L Hotamisligil GS Fatty acid binding proteins—the evolutionary crossroads of inflammatory and metabolic responses. J Nutr. 2004; 134:2464S-2468S 15333743 10.1093/jn/134.9.2464S PMC4027055

[41] Furuhashi M Tuncman G Gorgun CZ Makowski L Atsumi G Vaillancourt E Kono K Babaev VR Fazio S Linton MF Sulsky R Robl JA Parker RA Hotamisligil GS Treatment of diabetes and atherosclerosis by inhibiting fatty‐acid‐binding protein aP2. Nature. 2007; 447:959-965 17554340 10.1038/nature05844 PMC4076119

[42] von Eynatten M Breitling LP Roos M Baumann M Rothenbacher D Brenner H Circulating adipocyte fatty acid‐binding protein levels and cardiovascular morbidity and mortality in patients with coronary heart disease: a 10‐year prospective study. Arterioscler Thromb Vasc Biol. 2012; 32:2327-2335 22679309 10.1161/ATVBAHA.112.248609

